# Penta-O-galloyl-β-D-glucose induces G_1 _arrest and DNA replicative S-phase arrest independently of P21 cyclin-dependent kinase inhibitor 1A, P27 cyclin-dependent kinase inhibitor 1B and P53 in human breast cancer cells and is orally active against triple-negative xenograft growth

**DOI:** 10.1186/bcr2634

**Published:** 2010-09-01

**Authors:** Yubo Chai, Hyo-Jeong Lee, Ahmad Ali Shaik, Katai Nkhata, Chengguo Xing, Jinhui Zhang, Soo-Jin Jeong, Sung-Hoon Kim, Junxuan Lü

**Affiliations:** 1 The Hormel Institute, University of Minnesota, 801 16th Avenue NE, Austin, MN 55912, USA; 2 Cancer Preventive Material Development Research Center and Institute, College of Oriental Medicine, Kyung Hee University, 1 Hoegi-dong, Dongdaemun-gu, Seoul 131-701, Republic of Korea; 3 Department of Medicinal Chemistry, College of Pharmacy, University of Minnesota, 308 Harvard Street SE, Minneapolis, MN 55455, USA

## Abstract

**Introduction:**

Natural herbal compounds with novel actions different from existing breast cancer (BCa) treatment modalities are attractive for improving therapeutic efficacy and safety. We have recently shown that penta-1,2,3,4,6-O-galloyl-β-D-glucose (PGG) induced S-phase arrest in prostate cancer (PCa) cells through inhibiting DNA replicative synthesis and G_1 _arrest, in addition to inducing cell death at higher levels of exposure. We and others have shown that PGG through intraperitoneal (i.p.) injection exerts a strong *in vivo *growth suppression of human PCa xenograft models in athymic nude mice. This study aims to test the hypothesis that the novel targeting actions of PGG are applicable to BCa cells, especially those lacking proven drugable targets.

**Methods:**

Mono-layer cell culture models of p53-wild type estrogen receptor (ER)-dependent MCF-7 BCa cells and p53-mutant ER-/progesterone receptor (PR)- and Her2-regular (triple-negative) MDA-MB-231 BCa were exposed to PGG for a comprehensive investigation of cellular consequences and molecular targets/mediators. To test the *in vivo *efficacy, female athymic mice inoculated with MDA-MB-231 xenograft were treated with 20 mg PGG/kg body weight by daily gavage starting 4 days after cancer cell inoculation.

**Results:**

Exposure to PGG induced S-phase arrest in both cell lines as indicated by the lack of 5-bromo2'-deoxy-uridine (BrdU) incorporation into S-phase cells as well as G_1 _arrest. Higher levels of PGG induced more caspase-mediated apoptosis in MCF-7, in strong association with induction of P53 Ser^15 ^phosphorylation, than in MDA-MB-231 cells. The cell cycle arrests were achieved without an induction of cyclin dependent kinase (CDK) inhibitory proteins P21^Cip1 ^and P27^Kip1^. PGG treatment led to decreased cyclin D1 in both cell lines and over-expressing cyclin D1 attenuated G_1 _arrest and hastened S arrest. In serum-starvation synchronized MCF-7 cells, down-regulation of cyclin D1 was associated with de-phosphorylation of retinoblastoma (Rb) protein by PGG shortly before G_1_-S transition. *In vivo*, oral administration of PGG led to a greater than 60% inhibition of MDA-MB231 xenograft growth without adverse effect on host body weight.

**Conclusions:**

Our *in vitro *and *in vivo *data support PGG as a potential drug candidate for breast cancer with novel targeting actions, especially for a triple negative BCa xenograft model.

## Introduction

Breast cancer (BCa) is the major cause of cancer-related deaths for women in the US [1] and other Western countries. Approximately 60% to 70% of BCa cases express estrogen receptors (ERs) or progesterone receptors (PRs) or both, and another approximately 20% of cases have amplified HER-2 proto-oncogene and express high levels of the HER-2 protein [2]. Approximately 15% to 20% of BCa cases are in the category of triple-negative phenotype because of their lack of ER and PR and do not have amplification of HER-2 [2,3]. These patients have a very poor prognosis because, unlike the situation for other types of BCa, there is no clinically validated, molecularly targeted therapy. When surgical and radiation options are no longer applicable to these triple-negative patients, treatment with available cytotoxic and genotoxic chemotherapy drugs produces limited efficacy and significant side effects. There remains a strong and urgent need for safer anti-cancer compounds for the treatment/management of the triple-negative BCa and its metastasis. Novel agents with multiple targeting ability distinct from the known drugable targets could be useful for circumventing the limitations of current treatment options.

Penta-1,2,3,4,6-O-galloyl-β-D-glucose (PGG) is a naturally occurring gallotannin polyphenolic compound in Oriental herbs such as *Galla Rhois*, the gallnut of *Rhus chinensis *Mill, and the root of peony *Paeonia suffruticosa *Andrews [4]. A couple of earlier papers have examined the *in vitro *effects of PGG while using an ER^+ ^estrogen-dependent and p53-wild-type MCF-7 BCa cell culture model [5,6]. Chen and colleagues [5] reported that PGG induced G_1 _arrest in association with upregulated abundance of cyclin-dependent kinase inhibitor (CDKI) proteins 1A (p21^Cip1^) and 1B (p27^Kip1^). Later, the same group showed that PGG decreased ERα and the HER family of membrane tyrosine kinase (EGFR, HER-2, and HER-3) and PI3K/AKT signaling in MCF-7 cells [6]. A close inspection of the experimental designs of these studies revealed a lack of critical time-matched controls, and therefore the conclusions and the validity of the mechanistic work reported are questionable.

In cell culture studies, we recently showed that PGG induces caspase-mediated apoptosis in the human LNCaP prostate cancer (PCa) cells that express wild-type p53 [7]. The caspase-mediated apoptosis induction by PGG was mediated in large part by activation of p53 as established through siRNA (small interference RNA) knockdown and dominant negative mutant approaches [7]. More recently, we showed the induction of cell death with autophagic features (e.g., autophagosome formation and addition of a phosphatidylethanolamine moiety to the microtubule-associated protein 1 light chain 3 [LC3] to a faster moving LC3-II form on electrophoresis) by PGG of p53-null, PTEN-null, (high AKT) PC-3 PCa cells, which did not undergo caspase-mediated apoptosis after exposure to PGG [8]. We have also investigated the cell cycle effects of PGG in these and other PCa cells [9]. We showed for the first time that, irrespective of the p53 and androgen dependence status of the PCa cell lines, PGG exerted a rapid (within 2 hours) and potent (IC_50 _[half inhibitory concentration] of approximately 6 μM) inhibition of 5-bromo-2'-deoxy-uridine (BrdU) incorporation into S-phase cells. In isolated nuclei, PGG inhibited DNA replicative synthesis with an efficacy superior to that of a known DNA polymerase-alpha inhibitor, aphidocolin. We have found, in addition to the S-arrest action, a close association of downregulation of cyclin D1 with G_1 _arrest induced by PGG. Taken together, our data with PCa cells indicate that PGG induced S arrest, probably through DNA replicative blockage, and induced G_1 _arrest via cyclin D1 downregulation to contribute to its anti-cancer activity. These results sharply contrasted with the questionable BCa cell culture studies mentioned above [5,6]. Therefore, whether the S and G_1 _cell cycle arrests and caspase-mediated or autophagic cell death actions of PGG are applicable to BCa cells needs to be experimentally tested.

In addition to PCa cells, PGG was shown to induce G_1 _cell cycle arrest and apoptosis of leukemia [10,11] to inhibit invasion-related molecules such as matrix metalloprotease-9 in melanoma cells [12] and EGFR signaling [13] and VEGFR2 signaling and angiogenesis *in vitro *and *in vivo *[14], supporting multiple targeting actions. A number of *in vivo *studies by us and others in Lewis lung cancer allograft [14] and PCa xenograft [7,13] models with a dose of 20 or 25 mg/kg every other day have shown anti-cancer efficacy without adverse effect on body weight. These *in vivo *and *in vitro *studies suggest probable anti-cancer activity of PGG against BCa, especially triple-negative BCa. In this report, we evaluated the cell cycle and cell death actions of PGG against MDA-MB231 triple-negative BCa cells and MCF-7 BCa cells, and we established, for the first time, an impressive oral efficacy of PGG against xenograft growth established from human MDA-MB231 cells.

## Materials and methods

### Chemicals and reagents

PGG was prepared in-house by methanolysis of tannic acid in accordance with a published method [4,15]. The purity was approximately 99%. For treatment of cells in mono-layer culture, PGG was dissolved in dimethyl sulfoxide (DMSO) as a stock solution. The final DMSO added to cell culture medium was below 0.1%. Antibodies, including anti-CDK4, anti-P21^Cip1^, anti-P27^Kip1^, and anti-ERα, were purchased from Santa Cruz Biotechnology, Inc. (Santa Cruz, CA, USA). Additional antibodies specific for cleaved poly-ADP-ribose polymerase (cPARP) (p89), cyclin D1, p53-Ser^15^P, pRb-Ser^795^, pRb-Ser^807/811^, and pAKT(Ser^473^) were purchased from Cell Signaling Technology, Inc. (Danvers, MA, USA). Antibody for LC-3 was purchased from MBL International (Woburn, MA, USA).

### Cell culture and treatments

MCF-7 and MDA-MB231 cell lines were purchased from the American Type Culture Collection (Manassas, VA, USA). No cell line was derived directly from human tumor tissue for the purposes of this study. MCF-7 cells were grown in RPMI-1640 medium supplemented with 10% fetal bovine serum (FBS) without antibiotics in an incubator at 37°C with 5% CO_2_. MDA-MB231 cells were grown in L-15 medium supplemented with 10% FBS without antibiotics in an incubator at 37°C with atmospheric CO_2_. At 24 hours after plating, the medium was changed before starting the treatment with PGG or the other agents. To standardize all PGG/drug exposure conditions, cells were bathed in culture medium at a volume-to-surface area ratio of 0.2 mL/cm^2 ^(for example, 15 mL for a T75 flask and 5 mL for a T25 flask).

### Cell growth assay by crystal violet staining

For the evaluation of the overall inhibitory effect of PGG on cell number, the cells were treated with PGG daily (fresh medium) for 3 days. After treatment, the culture medium was removed and the cells were fixed in 1% glutaraldehyde solution in phosphate-buffered saline for 15 minutes. The fixed cells were stained with 0.02% aqueous solution of crystal violet for 30 minutes. After the washing with phosphate-buffered saline, the stained cells were solubilized with 70% ethanol. The absorbance at 570 nm with the reference filter at 405 nm was evaluated using a microplate reader (Beckman Coulter, Inc., Brea, CA, USA).

### BrdU incorporation and cell cycle measurement

The protocol was based on our previous publications [9,16]. After the desired experimental treatments, 10 μL of BrdU (9 mg/mL) solution was added to 5 mL of medium for 30 minutes before harvesting cells. The cells were collected by trypsinization, centrifuged at 1,600*g *for 6 minutes, fixed with 70% ethanol overnight, and analyzed for cell cycle distribution by propidium iodide/BrdU bivariate flow cytometry.

### Synchronic MCF-7 cell G_0_/G_1 _progression model

MCF-7 cells were seeded in RPMI-1640 medium supplemented with 10% FBS without antibiotics in an incubator at 37°C with 5% CO_2_. Twenty-four hours later, the cells were washed with serum-free phenol-red-free RPMI-1640 medium and then incubated in serum-free phenol-red-free medium for another 24 hours. One flask of cells was reserved as 0-hours baseline control. For the other flasks, serum-free medium was replaced with complete medium (10% FBS) to release cells from G_0 _arrest. At selected time points, cells were harvested for flow cytometry to analyze cell cycle distribution. For PGG treatment, cells were released into complete medium and treated simultaneously with serum stimulation or were exposed to PGG after different time periods of G_1 _progression until 24 hours, when the cells were collected for cell cycle analyses.

### Immunoblot analyses

The cell lysate was prepared in ice-cold lysis buffer as described previously [17]. Immunoblot analyses were essentially as described [17], except that the signals were detected by enhanced chemofluorescence with a Storm 840 scanner (Molecular Dynamics, now part of GE Healthcare, Little Chalfont, Buckinghamshire, UK).

### MDA-MB231 xenograft model

The animal use protocol was approved by the Kyung Hee University Institutional Animal Care and Use Committee and carried out at the Cancer Preventive Material Development Research Center, College of Oriental Medicine, Kyung Hee University (Seoul, South Korea). One million MDA-MB231 cells were mixed with 50% Matrigel (Becton, Dickinson and Company, Franklin Lakes, NJ, USA) and injected (in 100 μL) subcutaneously into the right flank of each 6-week-old female BALB/c athymic nude mouse (NARA Biotech, Deajon, South Korea). Starting 4 days after inoculation, 10 mice per group were given a daily gavage treatment of 2% Tween-80 (vehicle) or 20 mg of PGG per kg body weight. The dosage was based on our PCa xenograft work [7] and lung cancer allograft work [14]. Tumors were measured twice per week with a caliper, and tumor volume was calculated using the following formula: 1/2 (w1 × w2 × w2), where w1 represents the larger tumor diameter and w2 represents the smaller tumor diameter.

### Statistical analyses

Numerical data were expressed as mean ± standard error or standard deviation (where noted). Statistical analyses were carried out with GraphPad Prism (GraphPad Software, Inc., San Diego, CA, USA) software, and a *P *value of less than 0.05 was considered statistically significant. The data were analyzed by analysis of variance followed by Dunnett's multiple comparison post tests or other appropriate tests.

## Results

### PGG inhibited MCF-7 and MDA-MB231 breast cancer cell growth and induced caspase-mediated and caspase-independent cell death

**Figure 1:**
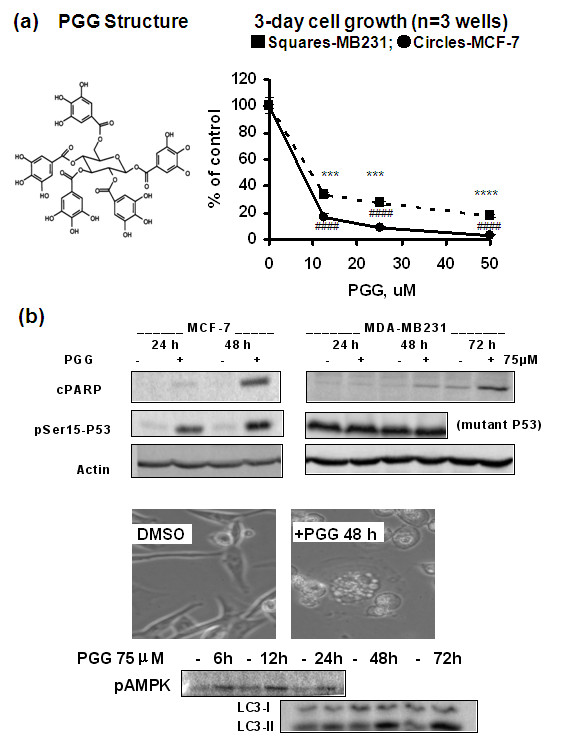
**Growth inhibitory and cell death actions of PGG in MCF-7 and MDA-MB231 cells**. **(a) **Overall inhibitory effects of PGG on MCF-7 and MDA-MB231 cell growth after 3 days of daily treatment with PGG in fresh medium. Values are mean ± standard error of the mean (*n *= 3 wells of 12-well plate). Statistical significance: ***P < 0.001; ****^,####^*P *< 0.0001 versus untreated control. Results are representative of two independent experiments. **(b) **Immunoblot detection of apoptotic cPARP (cleaved poly-ADP-ribose polymerase), P53-Ser^15 ^phosphorylation induced by PGG in MCF-7 or MDA-MB231 cells, and autophagy responses (pAMPK and LC3-II) in MDA-MB231 cells. Phase-contrast photomicrograph shows vaculolation typical of autophagy. The medium was not changed for PGG exposure of longer than 24 hours. DMSO, dimethyl sulfoxide; LC3, microtubule-associated protein 1 light chain 3; pAMPK, phospho-AMP kinase; PGG, penta-O-galloyl-β-D-glucose.

To evaluate the growth inhibitory effect on estrogen-dependent BCa cell line MCF-7 and the triple-negative BCa cell line MDA-MB231, we exposed these cells to daily changes of fresh complete medium with increasing concentrations of PGG. After 3 days of daily exposure, PGG decreased the number of MCF-7 and MDA-MB231 cells in a dose-dependent manner, and the IC_50 _of PGG for both cell lines was lower than 12.5 μM (Figure 1a). The p53-wild-type MCF-7 cells were more sensitive than the p53-mutant triple-negative MDA-MB231 cells to the growth suppression action of PGG at each tested concentration of PGG.

The difference in sensitivity of the two BCa cell lines was associated in part with the propensity for MCF-7 cells to undergo caspase-mediated apoptosis preceded by P53-Ser^15 ^phosphorylation (P53-Ser^15^P) (24 hours) and cleavage of PARP (48 hours) (Figure 1b). In the MDA-MB231 cells, whose mutant P53 phosphorylation was not responsive to PGG treatment, minimal cleavage of PARP (72 hours) was preceded by autophagic features as indicated morphologically by cytosolic vacuolation (48 hours); biochemically by an early (6 hours) increase of phosphorylation of AMPK (AMP-activated protein kinase), a well-known autophagy signaling kinase in response to nutrient deprivation [18]; and by increased phosphatidylethanolamine modification of the microtubule-associated protein 1 light chain 3 (LC3-I) to the faster moving LC-3II form (24 to 48 hours) (Figure 1b). These results therefore mirror our data (on PGG induction of apoptosis and other types of cell death) obtained with LNCaP [7] and PC-3 PCa [8] cells, respectively.

### PGG induced S and G_1 _arrests in MCF-7 and MDA-MB231 cells

Prompted by our findings of G_1 _and S arrests in different PCa cell lines (LNCaP, DU-145, and PC-3) [8,9], which contrasted with the reported G_1 _arrest in MCF-7 cells [5], we measured the cell cycle distribution patterns of MCF-7 and MDA-MB231 cells after exposure in complete medium to different concentrations of PGG for 6, 24, and 48 hours.

**Figure 2:**
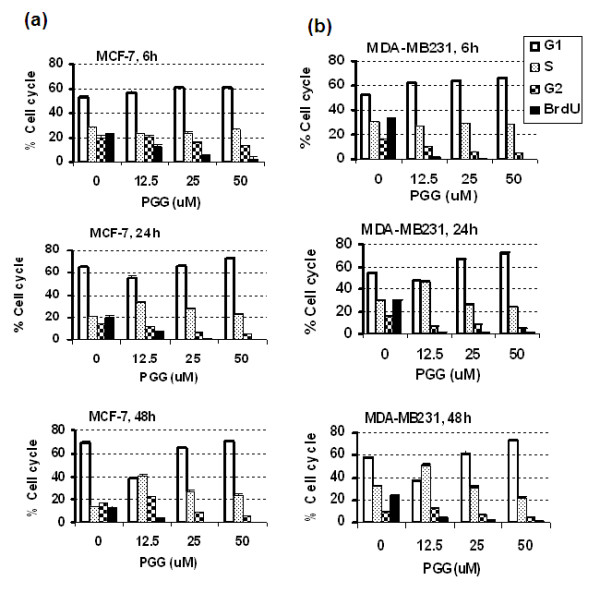
**The effect of PGG on cell cycle distribution of MCF-7 (a) and MDA-MB231 (b) cells detected by propidium iodide/BrdU-bivariate flow cytometric analyses**. Cells were exposed to increasing concentrations of PGG for 6, 24, and 48 hours. BrdU was added for the last 30 minutes to label S-phase cells active in DNA replication. Values are mean ± standard error of the mean (*n *= 4). Results are from two independent experiments with duplicate values at each concentration. The medium was not changed for PGG exposure of longer than 24 hours. Statistical significance: (a) BrdU incorporation at all three time points, one-way analysis of variance (ANOVA) *P *< 0.0001, with Dunnett's multiple comparison post test *P *value of less than 0.01 for 0 versus 12.5, 25, and 50 μM PGG. For G_1_, 6 hours *P *< 0.05 for 0 versus 25 and 50 μM PGG; 24 hours *P *< 0.01 for 0 versus 12.5 or 50 μM PGG; 48 hours *P *< 0.01 for 0 versus 12.5 μM PGG and *P *< 0.05 for 0 versus 50 μM PGG. For S, 24 hours/48 hours *P *< 0.01 for 0 versus 12.5 and 25 μM PGG. (b) BrdU incorporation at all three time points, one-way ANOVA *P *< 0.0001, with Dunnett's multiple comparison post test *P *value <0.01 for 0 versus 12.5, 25, and 50 μM PGG. For G_1_, 6 hours *P *< 0.05 for 0 versus 12.5, 25, and 50 μM PGG; 24 hours *P *< 0.01 for 0 versus 25 and 50 μM PGG; 48 hours *P *< 0.01 for 0 versus 50 μM PGG. For S, 24 hours/48 hours *P *< 0.01 for 0 versus 12.5 μM PGG. BrdU, 5-bromo-2'-deoxy-uridine; PGG, penta-O-galloyl-β-D-glucose.

In both MCF-7 and MDA-MB231 cells, PGG exposure for 6 hours led to a concentration-dependent increase of G_1_-phase cells and was accompanied by a decrease of G_2_-phase cells (Figure 2). Probably owing to their faster growth, the MB231 cells (Figure 2b) appeared to more readily achieve G_1 _arrest than the MCF-7 cells (Figure 2a) in the presence of the lowest PGG concentration tested. The percentage of S-phase cells remained relatively steady in both cell lines. Inspection of the BrdU incorporation index (measured, as we previously described, for 30 minutes of pulse labeling before cell harvest [9]) showed a near-complete blockage of DNA synthesis in the S-phase cells in MB231 cells at all three PGG exposure concentrations, whereas in the MCF-7 cells, a clear concentration dependency on PGG was observed.

For both cell lines, as time progressed to 24 and 48 hours, the lowest concentration of PGG (12.5 μM) was not able to hold the cells arrested in G_1_, manifesting as the accumulation of S-phase cells that remained incapable of incorporating BrdU. The higher concentrations of PGG (50 μM) kept cells arrested in G_1 _phase and S phase throughout the 6- to 48-hour period (Figure 2). The data therefore support both S arrest and G_1 _arrest by PGG in BCa cells, as in PCa cells [9].

### PGG did not alter P21^Cip1 ^and P27^Kip1 ^expression in breast cancer cells

An earlier report by Chen and colleagues [5] has claimed G_1 _arrest and P21^Cip1 ^and P27^Kip1 ^induction by PGG in MCF-7 cells, without including critical time-matched controls. We therefore examined these proteins as possible molecular mediators for the G_1 _and S arrests. Since we have reported the rapid P53-Ser^15^P by PGG treatment in LNCaP PCa cells [7] and have observed P53-Ser^15^P in PGG-exposed MCF-7 cells (Figure 1b) and since the P53-P21^Cip1 ^axis is best known for mediating G_1 _arrest by genotoxic stress [19], we focused on the relationship among these proteins in PGG-exposed MCF-7 cells.

**Figure 3:**
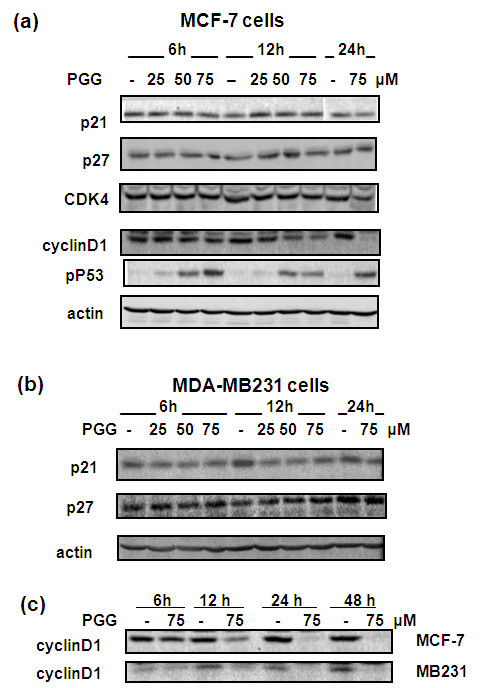
**Effect of PGG on cyclin D1, P21^Cip1^, and P27^Kip1 ^and other select cell cycle proteins in MCF-7 and MDA-MB231 cells detected by Western blot analyses**. **(a) **Cyclin D1, CDK4, P21^Cip1^, and P27^Kip1 ^expression and P53-Ser^15^P in MCF-7 cells. β-Actin was re-probed as loading control. **(b) **P21^Cip1 ^and P27^Kip1 ^expression in MDA-MB231 cells. **(c) **Time course of cyclin D1 expression in MCF-7 and MDA-MB231 cells treated with PGG from 12 to 48 hours. Patterns are representative of two experiments. The medium was not changed for PGG exposure of longer than 24 hours. P21^Cip1^, cyclin-dependent kinase inhibitor 1A; P27^Kip1^, cyclin-dependent kinase inhibitor 1B; PGG, penta-O-galloyl-β-D-glucose.

**Figure 4:**
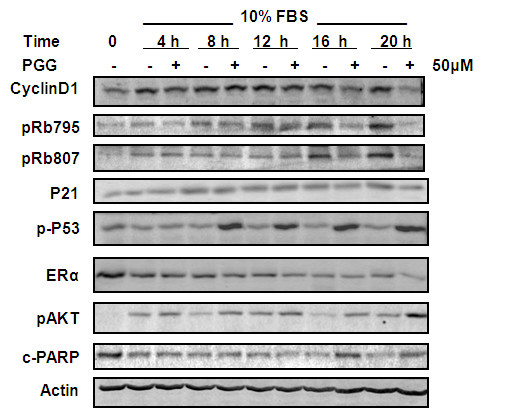
**The effect of PGG on cell cycle proteins in serum starvation-synchronized MCF-7 cells**. PGG was included at time of serum stimulation (as time 0). Western blot was used to detect Cyclin D1 expression and phosphorylation of Retinoblastoma protein, activation of P53-Ser^15^P, and expressions of P21^Cip1 ^and estrogen receptor-alpha. cPARP, cleaved poly-ADP-ribose polymerase; ERα, estrogen receptor-alpha; FBS, fetal bovine serum; P21^Cip1^, cyclin-dependent kinase inhibitor 1A; PGG, penta-O-galloyl-β-D-glucose.

We observed that PGG treatment activated P53-Ser^15^P at 6 hours with a clear concentration dependency but did not increase the protein abundance of either P21^Cip1 ^or P27^Kip1 ^(Figure 3a). Later, we found the same pattern of disengaged P53/P21^Cip1 ^response (that is, P53-Ser^15^P but not upregulated P21^Cip1^) in the synchronic MCF-7 model (Figure 4). In MDA-MB231 cells, we did not observe any induction of these two CDKI proteins by PGG (Figure 3b). These results suggest that PGG induced G_1 _arrest in the absence of detectable alterations of P21^Cip1 ^and P27^Kip1 ^protein abundance and was independent of P53 function in these BCa cells.

### PGG decreased cyclin D1 abundance in breast cancer cells

In contrast to a lack of expression change of P21^Cip1 ^or P27^Kip1^, PGG treatment significantly decreased the abundance of cyclin D1 in MCF-7 and MDA-MB231 cells (Figure 3a, c). PGG treatment decreased cyclin D1 expression as early as 6 hours, and by 12 hours, its expression decreased dramatically. From 24 to 48 hours, there was almost no detectable cyclin D1 expression in MCF-7 and MDA-MB231 cells treated with PGG at a high dose (Figure 3a, c).

**Figure 5:**
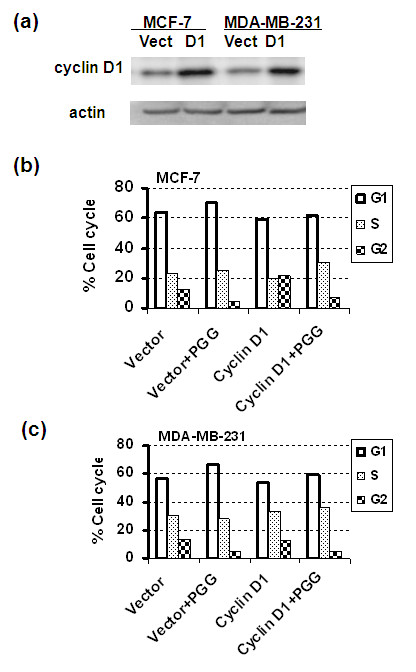
**Impact of overexpression of cyclin D1 on PGG-induced G_1 _arrest in MCF-7 and MDA-MB231 cells**. **(a) **Western blot verification of stable overexpression of cyclin D1 in MCF-7 and MDA-MB231 cells. **(b) **Cell cycle distribution of MCF-7 cells transfected with vector and cyclin D1 plasmid with or without PGG treatment for 24 hours. **(c) **Cell cycle distribution of MDA-MB231 cells transfected with vector and cyclin D1 plasmid with or without PGG treatment for 24 hours. Each bar reflects the average of two T25 flasks. The patterns are representative of two experiments. PGG, penta-O-galloyl-β-D-glucose.

To test the contribution of cyclin D1 downregulation to the G_1 _arrest, we made stable transfectants of MCF-7 and MDA-MB231 cells with forced overexpression of cyclin D1 (Figure 5a) (the expression plasmid was kindly provided by Joshua D Liao, The Hormel Institute, Austin, MN, USA). Compared with vector transfectant cells, the cyclin D1-overexpressing MCF-7 cells significantly attenuated PGG-induced G_1 _arrest (Figure 5b). Similarly, MDA-MB231 cells overexpressing cyclin D1 partially overcame PGG-induced G_1 _arrest (Figure 5c). Instead, PGG exposure of the cyclin D1-overexpressing cells hastened S arrest, without affecting G_2_-phase decline. The data suggest that G_1 _arrest and S arrest operate by independent mechanisms in BCa cells, as in PCa cells [9], and that cyclin D1 downregulation by PGG was an important contributor (but perhaps not the sole mediator) to G_1 _arrest.

### Defining G_1_-targeting action of PGG in a synchronic MCF-7 model

**Figure 6:**
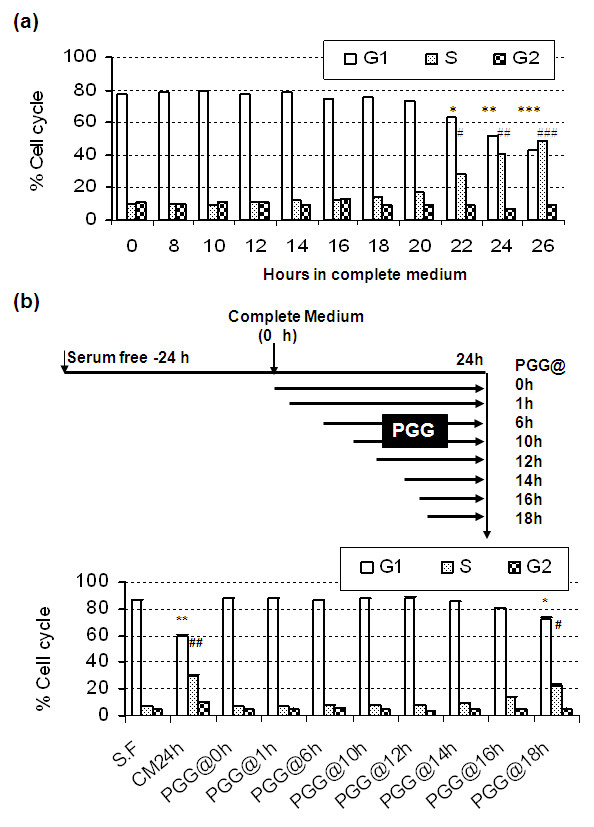
**Effect of PGG on G_0/1_-S progression in synchronized MCF-7 cells**. **(a) **The temporal kinetics of serum-stimulated progression of starvation-synchronized MCF-7 cells. Each time point was the average of duplicate flasks. *^,#^*P *< 0.05; **^,##^*P *< 0.01; ***^,###^*P *< 0.001 versus 0 time. **(b) **Impact of delaying PGG treatment with reference to serum stimulation on G_1 _arrest. Results are from two independent experiments with duplicate values at each time point. *^,#^*P *< 0.05; **^,##^*P *< 0.01 versus serum-free (SF) or PGG@0h-14 h. CM, complete medium; PGG, penta-O-galloyl-β-D-glucose.

To further probe the G_1_-targeting mechanisms of action of PGG without the complication from S arrest, we synchronized MCF-7 cells to G_0 _by serum starvation for 24 hours and released the cells into complete medium (this time point was referred to as 0 hours). Cell cycle distribution patterns suggested that the G_1_-phase cells started to transit into S phase between 20 and 22 hours of FBS re-stimulation (Figure 6a).

In this synchronic MCF-7 cell model, inclusion of PGG at the time of serum stimulation (PGG@0 hours) caused a complete block of G_1_-to-S transition, measured by flow cytometry at 24 hours (Figure 6b). To determine whether the presence of PGG during the early stage G_0/1 _progression was necessary for G_1 _arrest and to pinpoint the responsible molecular events, we delayed the starting time for PGG exposure in reference to serum stimulation. As shown in Figure 6b, delaying the starting exposure time to 14 hours (that is, PGG@14 hours) did not lessen the G_1_-arrest action of PGG. Starting PGG treatment at 16 to 18 hours was less able to prevent G_1_-to-S transition. These data indicated that the crucial time window for PGG targeting during G_1_/S progression was 16 to 18 hours after serum stimulation.

### PGG decreased cyclin D1 in synchrony with retinoblastoma de-phosphorylation in synchronic MCF-7 cells

In the synchronic MCF-7 model, serum stimulation led to increased cyclin D1 expression (4 hours was the earliest point sampled), which persisted through 20 hours (G_1_/S transit) (Figure 4). Serum stimulation increased survival signaling, as indicated by AKT phosphorylation, in a temporal pattern similar to that of cyclin D1 and suppressed background level apoptosis as indicated by the decreased cPARP. Serum stimulation decreased ERα, which declined progressively over time. A well-known downstream effector molecule of cyclin-CDK complexes for G_1 _progression is the retinoblastoma (Rb) protein [20]. Cyclin-CDK complexes phosphorylate Rb to decrease its binding to the E2F transcriptional factor, releasing E2F to activate expression of its target genes for G_1_/S transition. Indeed, we detected increased Rb phosphorylation at 12 hours at the Ser^795 ^site and 16 hours at Ser^807/811 ^sites prior to the onset of G_1_/S transition (20 hours).

Exposure of synchronic MCF-7 cells to PGG at the time of serum stimulation did not decrease cyclin D1 until 16 hours, coinciding with decreased Rb phosphorylation at Ser^795 ^and Ser^807/811 ^sites (Figure 4). Although P53-Ser^15^P was detected by 8 hours of PGG treatment, there was a clear absence of P21^Cip1 ^induction by PGG throughout 20 hours. Increased cPARP was detected by 16 hours, and this was preceded by increased AKT(Ser^473^) phosphorylation by several hours. PGG treatment did not affect ERα until the 20-hour time point. Given that P21^Cip1 ^abundance was not upregulated throughout the G_1 _phase by PGG, the data suggest that the G_1 _arrest was regulated predominantly by the cyclin D-CDK-Rb axis, preventing the release of E2F to promote the passage of the restriction point.

### Orally administered PGG suppresses MDA-MB231 breast cancer xenograft growth

**Figure 7:**
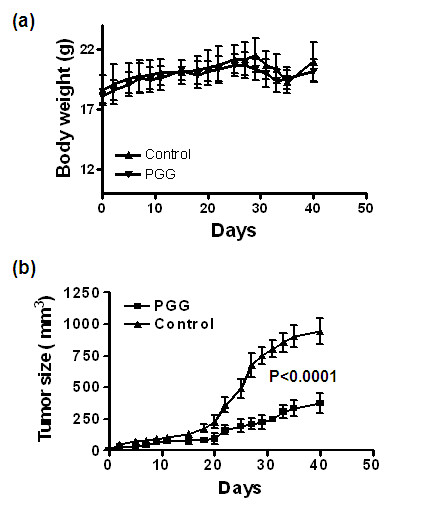
**PGG intake by oral gavage inhibits MDA-MB231 tumor growth in female athymic nude mice**. Starting 4 days after cell inoculation, PGG (20 mg/kg) was gavaged with 2% Tween-80 as vehicle to these animals once a day. **(a) **Body weight. **(b) **Tumor volume. Values are mean ± standard deviation (*n *= 10 mice per group). Statistical significance: analysis of variance PGG effect on tumor size, *P *< 0.0001. PGG, penta-O-galloyl-β-D-glucose.

The cell culture data presented above suggest probable *in vivo *anti-cancer efficacy of PGG against BCa growth. Because oral administration is the most practical and non-invasive way to deliver an anti-cancer agent, we evaluated the efficacy of PGG delivered by oral gavage against MDA-MB231 cells injected subcutaneously into the right flank of each female athymic nude mouse at the dosage of 20 mg/kg body weight, starting 4 days after cancer cell inoculation. This dosage of PGG did not exert any adverse effect on body weight of the host nude mice (Figure 7a). PGG treatment led to a significant inhibition of tumor growth rate over time (Figure 7b) and decreased the final tumor size by over 60% at necropsy.

## Discussion

As pointed out in the Introduction, there is an urgent clinical need for safe and effective treatment and preventive agents for triple-negative BCa. Our results presented above provide *in vitro *and *in vivo *data that support the potential for PGG to be such a promising drug candidate with multiple targeting actions, distinct from known drugable BCa targets such as the ER (for example, estrogen antagonist drug tamoxifen) and HER-2 (for example, inactivating monoclonal antibody herceptin). In cell culture, PGG treatment caused P53-Ser^15 ^phosphorylation (Figures 1, 3, and 4) and caspase-mediated apoptosis (Figures 1 and 4) in MCF-7 BCa cells. In p53-mutant MDA-MB231 triple-negative BCa cells, PGG caused not only apoptosis but also autophagic responses (Figure 1b). We showed that independently of P53 status or ERα status of the BCa cells, PGG induced S arrest and G_1 _arrest (Figure 2) without inducing P21^Cip1 ^and P27^Kip1 ^expression (Figures 3 and 4). Our data support cyclin D1 downregulation by PGG as an important mediating event for the G_1_-arrest action (Figures 3 to 5). The clear disengagement of P53-Ser^15 ^phosphorylation from the best-known P53 transcriptional target P21^Cip1 ^in PGG-exposed MCF-7 cells remains an interesting question for further investigation.

Our findings are important in two respects. First, they are consistent with recently published results for PCa cells [7-9], suggestive of a treatment applicability of PGG for cancers of other organ sites. The documented ability in this study to generate high-purity PGG in multi-gram quantities from tannic acid will enable us and others to explore the *in vivo *anti-cancer efficacy of PGG in relevant animal models of cancers of other organ sites. Second, the findings point out the possibility that some published data are highly questionable concerning the action mechanisms of PGG in BCa cells. In contrast to the data published by others [5], our data did not detect a change of P21^Cip1 ^and P27^Kip1 ^expression to be associated with the G_1_-arrest action of PGG (Figure 3). We also did not observe a dramatic impact of PGG on ERα abundance or a suppression of AKT phosphorylation (Figure 4), as were claimed [6]. Instead, PGG treatment increased AKT phosphorylation in MCF-7 cells (Figure 4), as we have reported for a similar increase of AKT phosphorylation in PC-3 cells by PGG [8]. Although many reasons could be cited for the discrepancies between our data and the previous reports [5,6], their lack of time-matched controls could be the leading cause of confusion and misleading conclusions.

Our *in vivo *data demonstrated, for the first time, a growth inhibitory efficacy of PGG against triple-negative BCa and supported the oral bioavailability of PGG. The potency of PGG (20 mg per kg body weight) is remarkable, especially considering that PGG was given by the oral route. Furthermore, just the fact that PGG is orally available and therefore can be self-administered by patients will have a major impact on reducing the health-care delivery cost compared with injection-only drugs (such as paclitaxel) that have to be given by health-care professionals. The data on efficacy and safety of PGG provide an impetus for further studies about the therapeutic application of PGG and its *in vivo *molecular targets and mechanisms of action.

## Conclusions

Our cell culture data showed that PGG could induce both G_1 _and S arrests in BCa cells, regardless of their ER or P53 functional status. Cyclin D1 downregulation by PGG was a mechanism for G_1 _arrest in BCa cell lines, and the data ruled out P21^Cip1 ^and P27^Kip1 ^for mediating G_1 _arrest. We demonstrated for the first time that PGG given by oral administration was quite safe to the host nude mice and potent for suppressing a triple-negative BCa xenograft model. The therapeutic and chemopreventive utility of PGG for BCa merits further study.

## Abbreviations

BCA: breast cancer; BRDU: 5-bromo-2'-deoxy-uridine; CDK: cyclin-dependent kinase; CDKI: cyclin-dependent kinase inhibitor; CPARP: cleaved poly-ADP-ribose polymerase; DMSO: dimethyl sulfoxide; ER: estrogen receptor; FBS: fetal bovine serum; IC_50_: half inhibitory concentration; LC3: microtubule-associated protein 1 light chain 3; PCA: prostate cancer; PGG: penta-O-galloyl-β-D-glucose; PR: progesterone receptor; P21^Cip1^: cyclin-dependent kinase inhibitor 1A; P27^Kip1^: cyclin-dependent kinase inhibitor 1B; RB: retinoblastoma.

## Competing interests

The authors declare that they have no competing interests.

## Authors' contributions

JL conceived of and coordinated the studies, designed the experiments, and drafted and edited the manuscript. SHK helped to conceive of and coordinate the studies and to design the experiments. YC helped to perform cell culture experiments and statistical analyses, and to draft the manuscript. HJL helped to design the experiments, to carry out the xenograft study, and to draft the manuscript. JZ helped to design the experiments and to perform cell culture experiments. KN helped to perform cell culture experiments and statistical analyses. SJJ helped to carry out the xenograft study. AAS and CX scaled-up PGG preparation from tannic acid and performed chemical characterization. All authors read and approved the final manuscript.

## References

[B1] Jemal A Siegel R Xu J Ward E Cancer Statistics, 2010 CA Cancer J Clin 2010 in press 20610543 10.3322/caac.20073

[B2] Brenton JD Carey LA Ahmed AA Caldas C Molecular classification and molecular forecasting of breast cancer: ready for clinical application? J Clin Oncol 2005 23 7350 7360 10.1200/JCO.2005.03.3845 16145060

[B3] Rahman M Davis SR Pumphrey JG Bao J Nau MM Meltzer PS Lipkowitz S TRAIL induces apoptosis in triple-negative breast cancer cells with a mesenchymal phenotype Breast Cancer Res Treat 2009 113 217 230 10.1007/s10549-008-9924-5 18266105 PMC2615075

[B4] Zhang J Li L Kim SH Hagerman AE Lu J Anti-cancer, anti-diabetic and other pharmacologic and biological activities of penta-galloyl-glucose Pharm Res 2009 26 2066 2080 10.1007/s11095-009-9932-0 19575286 PMC2822717

[B5] Chen WJ Chang CY Lin JK Induction of G1 phase arrest in MCF human breast cancer cells by pentagalloylglucose through the down-regulation of CDK4 and CDK2 activities and up-regulation of the CDK inhibitors p27(Kip) and p21(Cip) Biochem Pharmacol 2003 65 1777 1785 12781329 10.1016/s0006-2952(03)00156-4

[B6] Hua KT Way TD Lin JK Pentagalloylglucose inhibits estrogen receptor alpha by lysosome-dependent depletion and modulates ErbB/PI3K/Akt pathway in human breast cancer MCF-7 cells Mol Carcinog 2006 45 551 560 10.1002/mc.20226 16637063

[B7] Hu H Lee HJ Jiang C Zhang J Wang L Zhao Y Xiang Q Lee EO Kim SH Lu J Penta-1,2,3,4,6-O-galloyl-beta-D-glucose induces p53 and inhibits STAT3 in prostate cancer cells *in vitro *and suppresses prostate xenograft tumor growth *in vivo* Mol Cancer Ther 2008 7 2681 2691 10.1158/1535-7163.MCT-08-0456 18790750

[B8] Hu H Chai Y Wang L Zhang J Lee HJ Kim SH Lu J Pentagalloylglucose induces autophagy and caspase-independent programmed deaths in human PC-3 and mouse TRAMP-C2 prostate cancer cells Mol Cancer Ther 2009 8 2833 2843 10.1158/1535-7163.MCT-09-0288 19825802 PMC2838500

[B9] Hu H Zhang J Lee HJ Kim SH Lu J Penta-O-galloyl-beta-D-glucose induces S- and G(1)-cell cycle arrests in prostate cancer cells targeting DNA replication and cyclin D1 Carcinogenesis 2009 30 818 823 10.1093/carcin/bgp059 19269999 PMC2675654

[B10] Pan MH Lin JH Lin-Shiau SY Lin JK Induction of apoptosis by penta-O-galloyl-beta-D-glucose through activation of caspase-3 in human leukemia HL-60 cells Eur J Pharmacol 1999 381 171 183 10.1016/S0014-2999(99)00549-X 10554885

[B11] Chen WJ Lin JK Induction of G1 arrest and apoptosis in human jurkat T cells by pentagalloylglucose through inhibiting proteasome activity and elevating p27Kip1, p21Cip1/WAF1, and Bax proteins J Biol Chem 2004 279 13496 13505 10.1074/jbc.M212390200 14726525

[B12] Ho LL Chen WJ Lin-Shiau SY Lin JK Penta-O-galloyl-beta-D-glucose inhibits the invasion of mouse melanoma by suppressing metalloproteinase-9 through down-regulation of activator protein-1 Eur J Pharmacol 2002 453 149 158 10.1016/S0014-2999(02)02340-3 12398898

[B13] Kuo PT Lin TP Liu LC Huang CH Lin JK Kao JY Way TD Penta-O-galloyl-beta-D-glucose suppresses prostate cancer bone metastasis by transcriptionally repressing EGF-induced MMP-9 expression J Agric Food Chem 2009 57 3331 3339 10.1021/jf803725h 19320436

[B14] Huh JE Lee EO Kim MS Kang KS Kim CH Cha BC Surh YJ Kim SH Penta-O-galloyl-beta-D-glucose suppresses tumor growth via inhibition of angiogenesis and stimulation of apoptosis: roles of cyclooxygenase-2 and mitogen-activated protein kinase pathways Carcinogenesis 2005 26 1436 1445 10.1093/carcin/bgi097 15845650

[B15] Chen Y Hagerman AE Characterization of soluble non-covalent complexes between bovine serum albumin and beta-1,2,3,4,6-penta-O-galloyl-D-glucopyranose by MALDI-TOF MS J Agric Food Chem 2004 52 4008 4011 10.1021/jf035536t 15186130

[B16] Malewicz B Wang Z Jiang C Guo J Cleary MP Grande JP Lu J Enhancement of mammary carcinogenesis in two rodent models by silymarin dietary supplements Carcinogenesis 2006 27 1739 1747 10.1093/carcin/bgl032 16597642

[B17] Jiang C Wang Z Ganther H Lu J Caspases as key executors of methyl selenium-induced apoptosis (anoikis) of DU-145 prostate cancer cells Cancer Res 2001 61 3062 3070 11306488

[B18] Puissant A Robert G Fenouille N Luciano F Cassuto JP Raynaud S Auberger P Resveratrol promotes autophagic cell death in chronic myelogenous leukemia cells via JNK-mediated p62/SQSTM1 expression and AMPK activation Cancer Res 2010 70 1042 1052 10.1158/0008-5472.CAN-09-3537 20103647

[B19] Dulić V Kaufmann WK Wilson SJ Tlsty TD Lees E Harper JW Elledge SJ Reed SI p53-dependent inhibition of cyclin-dependent kinase activities in human fibroblasts during radiation-induced G1 arrest Cell 1994 76 1013 1023 10.1016/0092-8674(94)90379-4 8137420

[B20] Prall OW Sarcevic B Musgrove EA Watts CK Sutherland RL Estrogen-induced activation of Cdk4 and Cdk2 during G1-S phase progression is accompanied by increased cyclin D1 expression and decreased cyclin-dependent kinase inhibitor association with cyclin E-Cdk2 J Biol Chem 1997 272 10882 10894 10.1074/jbc.272.16.10882 9099745

